# MAL expression downregulation through suppressive H3K27me3 marks at the promoter in HPV16-related cervical cancers is prognostically relevant and manifested by the interplay of novel MAL antisense long noncoding RNA AC103563.8, E7 oncoprotein and EZH2

**DOI:** 10.1186/s13148-024-01651-9

**Published:** 2024-03-10

**Authors:** Abarna Sinha, Abhisikta Ghosh, Arnab Ghosh, Sonia Mathai, Jaydip Bhaumik, Asima Mukhopadhyay, Arindam Maitra, Nidhan K. Biswas, Sharmila Sengupta

**Affiliations:** 1 National Institute of Biomedical Genomics, P.O.: N.S.S, Kalyani, 741251 West Bengal India; 2 Tata Medical Center, Kolkata, West Bengal India; 3 Kolkata Gynecological Oncology Trials and Translational Research Group, Kolkata, West Bengal India

**Keywords:** Antisense long noncoding RNA AC103563.8, Cervical cancer, EZH2-mediated H3K27me3 marks, HPV16-E7, MAL, Patient survival

## Abstract

**Background:**

MAL (T-lymphocyte maturation-associated protein) is highly downregulated in most cancers, including cervical cancer (CaCx), attributable to promoter hypermethylation. Long noncoding RNA genes (lncGs) play pivotal roles in CaCx pathogenesis, by interacting with human papillomavirus (HPV)-encoded oncoproteins, and epigenetically regulating coding gene expression. Hence, we attempted to decipher the impact and underlying mechanisms of MAL downregulation in HPV16-related CaCx pathogenesis, by interrogating the interactive roles of MAL antisense lncRNA AC103563.8, E7 oncoprotein and PRC2 complex protein, EZH2.

**Results:**

Employing strand-specific RNA-sequencing, we confirmed the downregulated expression of MAL in association with poor overall survival of CaCx patients bearing HPV16, along with its antisense long noncoding RNA (lncRNA) AC103563.8. The strength of positive correlation between MAL and AC103563.8 was significantly high among patients compared to normal individuals. While downregulated expression of MAL was significantly associated with poor overall survival of CaCx patients bearing HPV16, AC103563.8 did not reveal any such association. We confirmed the enrichment of chromatin suppressive mark, H3K27me3 at MAL promoter, using ChIP-qPCR in HPV16-positive SiHa cells. Subsequent E7 knockdown in such cells significantly increased MAL expression, concomitant with decreased EZH2 expression and H3K27me3 marks at MAL promoter. In silico analysis revealed that both E7 and EZH2 bear the potential of interacting with AC103563.8, at the same binding domain. RNA immunoprecipitation with anti-EZH2 and anti-E7 antibodies, respectively, and subsequent quantitative PCR analysis in E7-silenced and unperturbed SiHa cells confirmed the interaction of AC103563.8 with EZH2 and E7, respectively. Apparently, AC103563.8 seems to preclude EZH2 and bind with E7, failing to block EZH2 function in patients. Thereby, enhanced EZH2 expression in the presence of E7 could potentially inactivate the MAL promoter through H3K27me3 marks, corroborating our previous results of MAL expression downregulation in patients.

**Conclusion:**

AC103563.8-E7-EZH2 axis, therefore, appears to crucially regulate the expression of MAL, through chromatin inactivation in HPV16-CaCx pathogenesis, warranting therapeutic strategy development.

**Supplementary Information:**

The online version contains supplementary material available at 10.1186/s13148-024-01651-9.

## Background

Cervical cancer (CaCx) is the second most common cancer among Indian women with high mortality rates (https://gco.iarc.fr/today/data/factsheets/populations/356-india-fact-sheets.pdf). Persistent infection with human papillomavirus (HPV) is the major aetiological factor for the development of cervical cancer [1]. HPV16 is the most predominant type in India. It is already well established that at the molecular level, viral oncoprotein E6 and E7 are the major transforming agents that drive CaCx development. E7 is established to be much more conserved than E6 [2] and hence is considered as the most crucial oncoprotein. E7 protein interacts with host protein pRb and others [3] to enhance cell cycle progression leading to cancer.

HPV infection, which is sexually transmitted, gets cleared among majority of such sexually active women within a period of two years [4]. Only those women who develop persistent infection, appear to be at risk of CaCx. This often happens through development of progressive cervical lesions, such as low-grade intraepithelial lesions (LSIL), high-grade intraepithelial lesions (HSIL) and CaCx over a period of approximately twenty years [5].

In the developed countries, vaccination, active population screening, proper diagnostic and therapeutic advances including targeted therapy and immunotherapy have been significant in curbing CaCx development. Unfortunately, in India, it remains to be one of the most common sources of cancer-related deaths among women [6, 7], attributable mostly to late-stage diagnosis and therapy failure. In this context, the primary requirement appears to be identification of suitable diagnostic and prognostic markers, to identify specific targets for therapy. Of late, evidences highlight that noncoding RNAs are capable of regulating biological and pathological processes that manifest in various diseases, including cancers [8]. Recent studies have shown that lncRNAs can epigenetically or post-transcriptionally regulate coding gene transcription, involving alternative splicing mechanisms [9]. Current evidences show that long noncoding RNAs (lncRNAs) play an important regulatory role in cancer pathogenesis, including progression, response to therapy, and prognosis [10, 11], through their influence on many cancer-associated hallmarks [12]. A recent study has revealed interactions between viral oncoproteins and lncRNAs [13], besides a study from our laboratory showing the interaction between HPV16 E7 oncoprotein and lncRNA HOTAIR [14]. Such findings therefore, establish the significant functional roles of lncRNAs in CaCx pathogenesis [12]. Hence, exploration of lncRNAs-mRNAs-viral oncoprotein axes merit attention, with the potential of novel biomarker or target discovery for the HPV-driven CaCx cases.

A number of evidences have established that long noncoding RNAs (lncRNAs) play a crucial role in regulating gene expression and ultimately causing cancer [10]. LncRNAs, epigenetically regulate gene expression, bind and inactivate miRNAs through a sponging effect, regulate mRNA stability, and support macromolecular complex assembly through scaffold formation [11]. A proportion of lncRNAs are Natural Antisense Transcripts (ncNATs), which can regulate the expression of their sense coding genes, at the same locus, by various mechanisms [15, 16]. Some recent genome wide studies have shown that coding transcripts and their antisense transcripts are associated with cancer and are correlated in their expression [(17, 18)]. Advanced RNA-seq technology, strand-specific RNA sequencing (ssRNA-seq), has opened the door for lncRNA research world-wide.

In this study, we chose to focus on T lymphocyte maturation-associated protein (MAL) and its novel ncNAT AC103563.8. This gene pair was highly downregulated and showed significantly enhanced correlation within HPV16-positive CaCx cases, in comparison to healthy individuals, as identified employing ss-RNA seq in our previous study [19]. MAL is a known biomarker for CaCx [20–22]. It is also known that hypermethylation of MAL promoter increases with disease severity [23]. There are various reports which show that methylation levels of CADM1 and MAL, are used for triage, among CaCx patients [23–28]. The combination of HPV testing along with these methylation markers are useful as an effective molecular screening strategy for HPV-positive patients. Therefore, based on the relevance of MAL in CaCx pathogenesis [22, 29], we undertook further analyses to uncover the interplay between HPV16 E7 oncoprotein, host molecules MAL, AC103563.8 and EZH2, a PRC2 complex member, which is known to create chromatin suppression marks and also facilitate promoter hypermethylation [30]. Our findings strongly justify the biological relevance of correlated co-expression of antisense lncRNAs, with the sense genes and the potential role of the AC103563.8/E7/EZH2 axis in MAL deregulated expression, unveiling a novel path for exploring therapeutics of HPV16-related CaCx.

## Results

### Significant downregulated expression of MAL and MAL antisense lncRNA AC103563.8 in HPV16-positive CaCx patients along with significant correlated co-expression between the two

MAL is located on chromosome 2: 95,691,422–95,719,737 in the forward strand. AC103563.8 is also located on chromosome 2: 95,690,938–95,692,454, but in the reverse strand (Additional file 1: Figure S1). A small overlap is also present between these two genes in the opposite strands. Taken together, the lncRNA AC103563.8 appears to be antisense to MAL. This is further justified based on our findings provided below.

Considering strand specific RNA-seq analysis of 44 HPV16-positive CaCx patients and 34 HPV negative normal healthy individuals [19], we identified the DEGs and considered only those that were encoded from the genic regions, in this study. Thus, focussing on only the DEcGs and DElncGs (antisense and sense intronic), we identified several significantly correlated DEcGs and DElncGs, which included the pair MAL and its antisense AC103563.8. This pair was one out of 17 such gene pairs, which further revealed increased correlative strength among the patients, as compared to the normal individuals. Thus, along with the availability of reports highlighting MAL downregulation, concomitant with MAL promoter hypermethylation as a biomarker of CaCx [22, 28], we proceeded to further unfold the biological and clinical relevance of MAL downregulation in HPV16-positive CaCx patients.

**Fig. 1:**
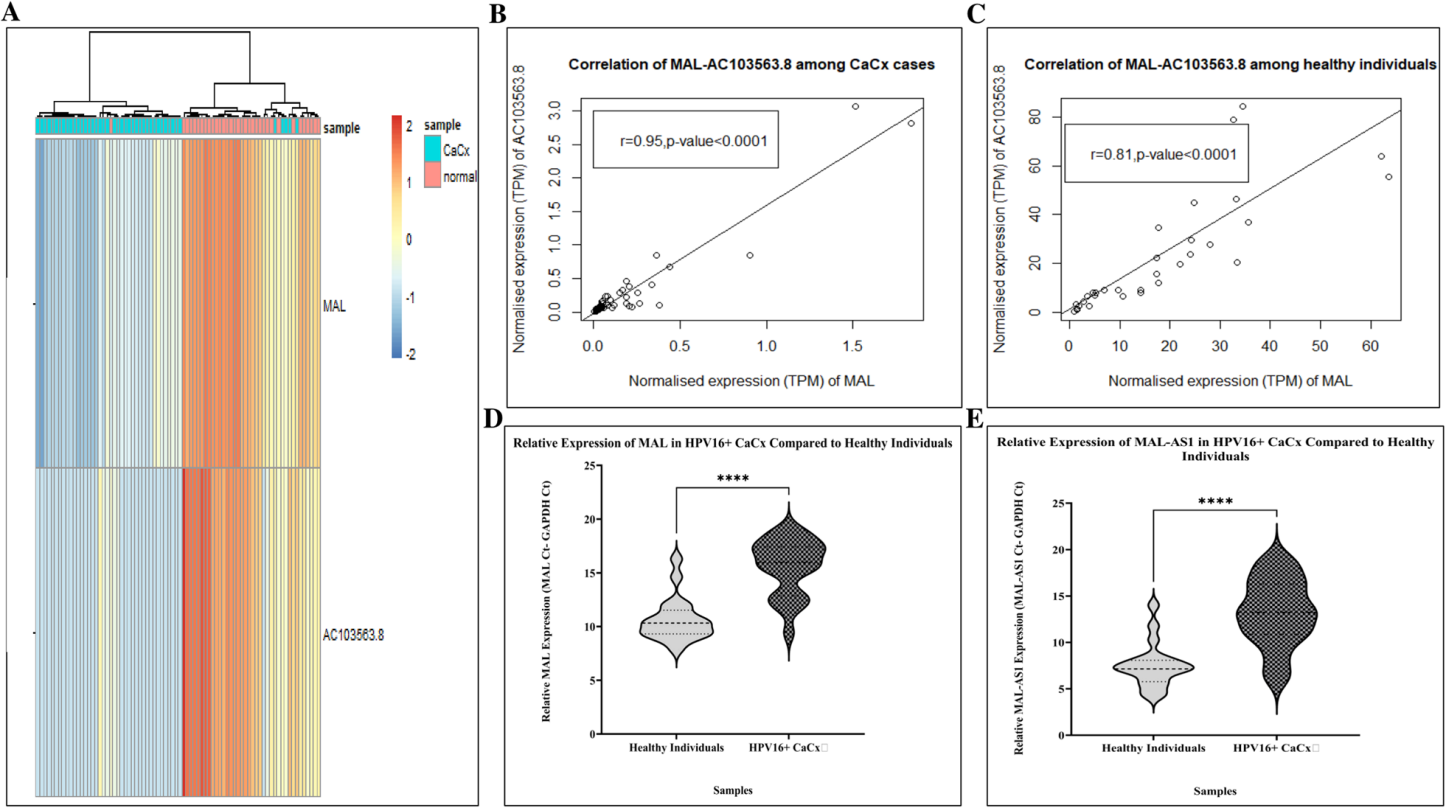
Visualisation of the expression and correlative relationship of the DEG pair: MAL- AC103563.8. **A** Unsupervised hierarchical clustering demarcating the CaCx patients from the normal individuals based on MAL and AC103563.8 gene expression profiles. The heatmap represents normalised counts, which were log2 transformed after adding constant 1 to all values of genes. The rows represent the gene names, and the columns represent the samples (blue bar represents the CaCx patient samples and pink bar represents the samples from normal individuals). Pearson’s correlation coefficient of MAL and AC103563.8 among (**B**) CaCx cases and (**C**) normal healthy individuals, respectively. Real-time qPCR based relative expression of **D** MAL and **E** AC103563.8 (MAL-AS1) in an additional cohort of HPV16-positive CaCx patients compared to healthy individuals. Relative gene expression reflects expression of the gene minus expression of the house-keeping gene (GAPDH), and is inversely proportional to the fold change. Thus, higher relative gene expression designates lower fold change. The data are represented as mean ± SD

We identified that MAL (|log2(FC)|= −6.8, FDR corrected *p* < 1.28E−63) and its ncNAT AC103563.8 (|log2(FC)|= −3.8, FDR corrected *p* < 3.61E−17) were both highly downregulated among the patients (Fig. 1A). We found the expression of MAL and AC103563.8 were positively correlated at the transcript level among the patients (*r* = 0.95, *p* = 2.04e−23) as well as in normal healthy individuals (*r* = 0.81, *p* = 4.093482e−09), as calculated from the TPM data obtained from RNA-sequencing (Fig. 1B and C). The correlation suggests that the antisense counterpart of MAL could probably play a role in the regulation of the expression of its coding gene counterpart MAL. Also, the correlative strength of this gene-pair was stronger among the CaCx patients, as opposed to the normal individuals, based on the difference in correlation coefficient of the two groups (*z* = −3.02, *p* = 0.0025).

Further, we validated the expression status of this gene-pair by real-time qPCR-based assay, employing an additional sample cohort of HPV16-positive CaCx patients (*n* = 22) and HPV negative healthy individuals (*n* = 19) (Fig. 1D-E). Significant downregulated expression of both MAL (0.03-fold, *p* < 0.0001) and AC103563.8 (0.019-fold, *p* < 0.0001) was found among HPV16-positive CaCx patients compared to normal individuals.

### Association of low expression of MAL with poor overall patient survival

**Fig. 2:**
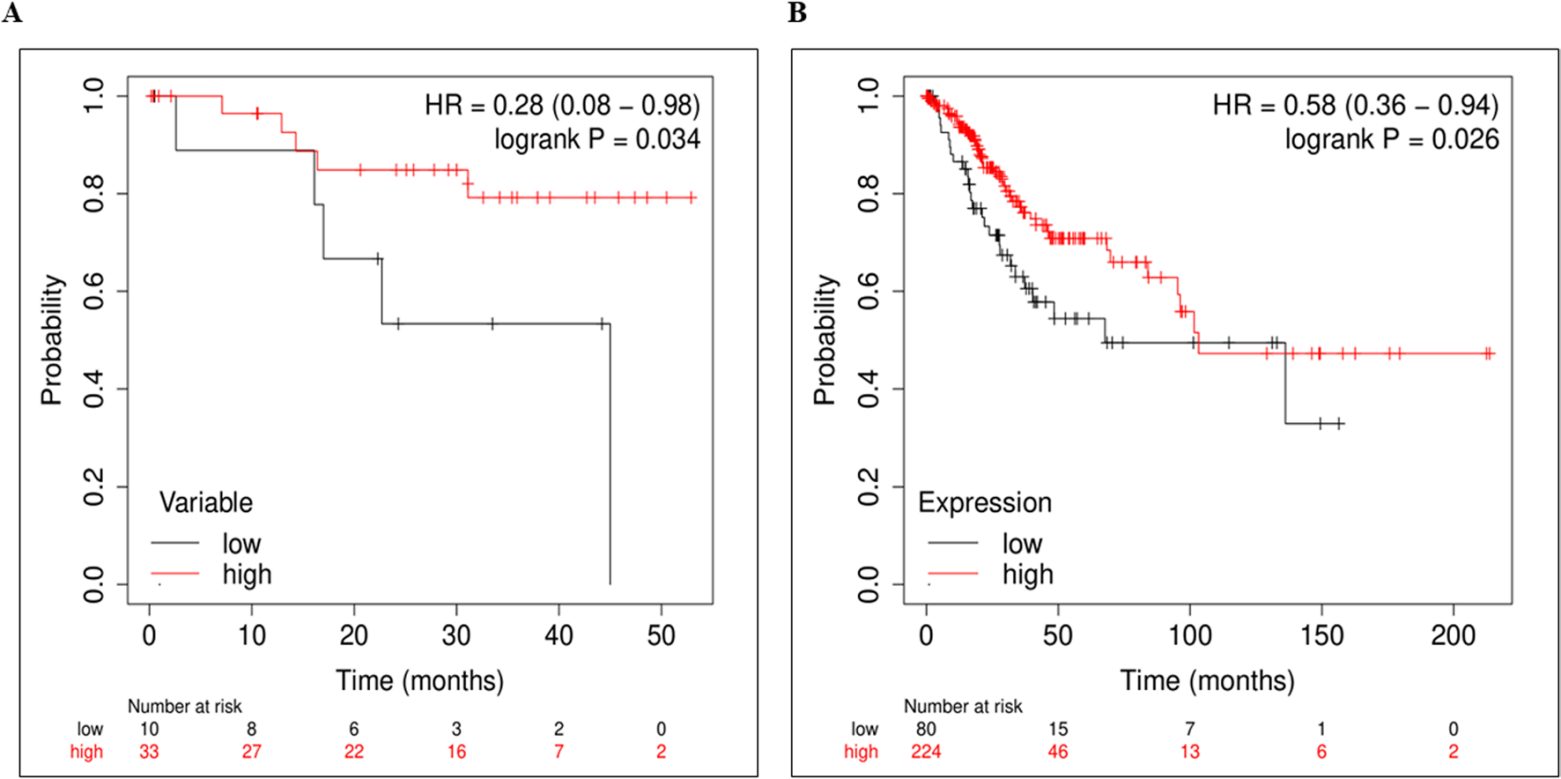
Kaplan–Meier survival analysis of MAL. KM plotter showed MAL to be significantly associated with poor patient overall-survival at low expression in (**A**) our cohort and in (**B**) TCGA-CESC cohort

To assess the relevance of MAL in the prognosis of patients, we determined the association of MAL expression with patient overall-survival, employing Kaplan–Meier survival analysis. For this analysis, we considered the RNA-seq data of our cohort, which had the follow-up data for approximately 4 years, along with the TCGA-CESC patient cohort survival data. We identified low expression of MAL to be significantly associated with poor patient prognosis, i.e. overall survival of patients in both the patient cohorts (Fig. 2A and B, respectively). Thereby, downregulated expression of MAL appears to be of significant clinical and prognostic relevance in case of patients with CaCx.

### HPV16-E7 oncoprotein-mediated enrichment of H3K27me3 chromatin suppressive marks at MAL gene promoter of SiHa cells, concomitant with enhanced EZH2 expression and decreased MAL expression

An earlier study [20] on CaCx pathogenesis identified that hypermethylation of MAL promoter was proportional to disease severity. A previous study from our laboratory also confirmed that the viral oncoprotein E7 interacts with the long noncoding RNA HOTAIR to epigenetically reprogram the promoter regions of genes, thereby modifying their expression profiles in HPV16-related CaCx [14]. Together, such findings prompted us to decipher the association of chromatin suppressive mark, H3K27me3, in the promoter region of MAL, with MAL gene expression downregulation, in the presence of E7.

**Fig. 3:**
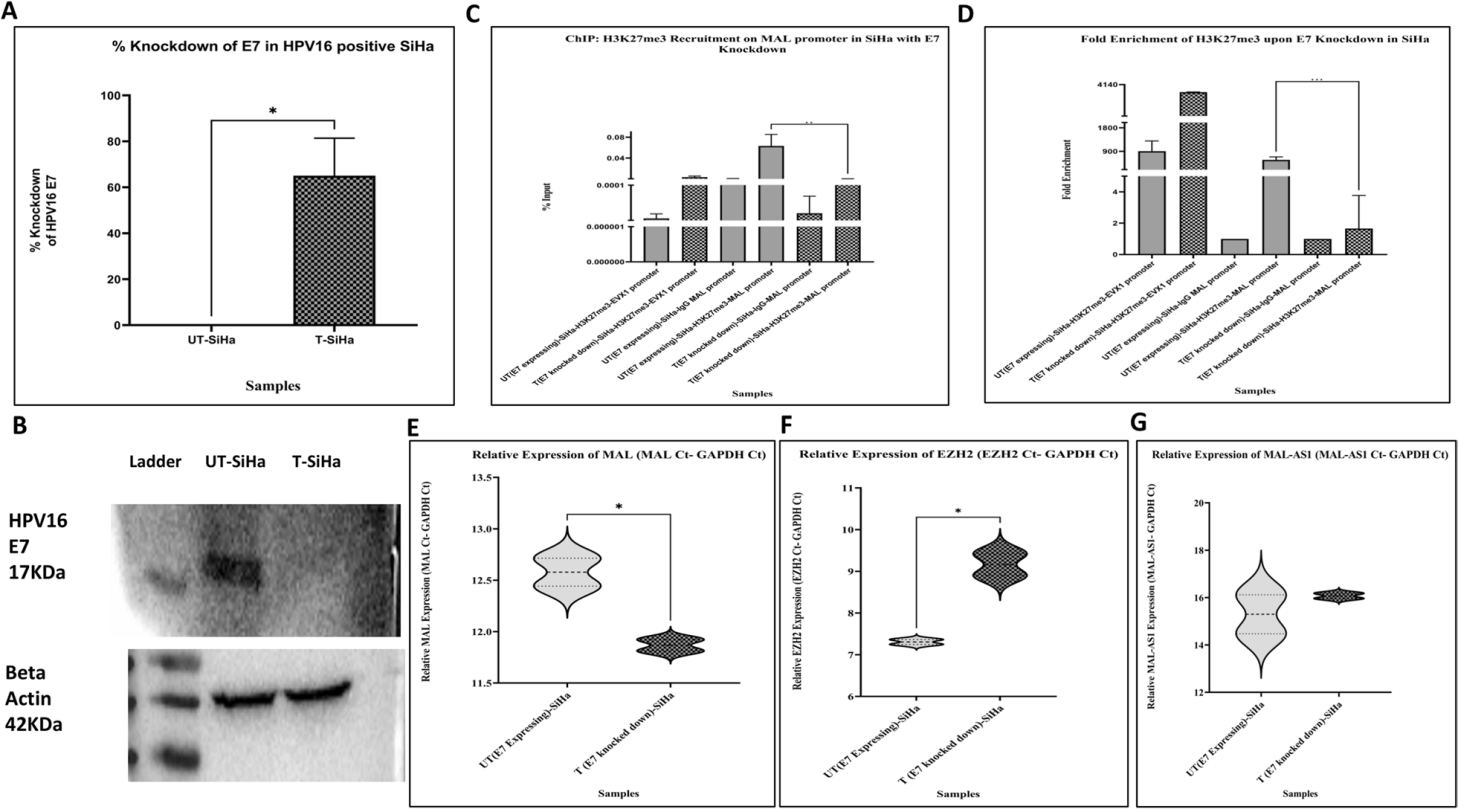
Knockdown of E7 by siRNA against E7 in HPV16-positive CaCx cell line, SiHa. **A** TaqMan-based qRT-PCR revealed 69% knockdown in HPV16 E7 expression in transfected group (T-SiHa) compared to the untransfected (UT-SiHa). **B** Upper panel: Western blot showing knockdown of E7 protein (19KDa) in transfected group (T-SiHa) compared to the untransfected (UT-SiHa). Lower panel: Western blot performed for house-keeping protein β-actin (42 KDa) for UT-SiHa (untransfected SiHa) and T-SiHa (transfected SiHa). The data are represented as mean ± SD from two independent experiments. Chromatin immunoprecipitation (ChIP)-qPCR with H3K27me3 antibody (**C, D**). **C** Fold enrichment showing the depletion of H3K27me3 at the MAL promoter in the transfected (T SiHa) when compared to that of untransfected (UT SiHa). EVX1 promoter is shown as the positive control for H3K27me3 in UT and T SiHa. The data are represented as mean ± SD from three independent experiments. **D** % input showing the depletion of H3K27me3 at the MAL promoter in transfected (T SiHa) when compared to that of untransfected (UT SiHa). Effect of E7 knockdown in HPV16-positive SiHa cells (E–G). **E** Relative expression of MAL **F** EZH2 **G** AC103563.8, respectively, in transfected (T SiHa) when compared to that of untransfected (UT SiHa). These plots depict the relative gene expression, i.e. expression of the gene—expression of the house-keeping gene (GAPDH). Relative gene expression is inversely proportional to the fold change. Thus, higher relative gene expression designates lower fold change. The data are represented as mean ± SD from two independent experiments

We employed HPV16-positive CaCx cell line SiHa to knockdown the expression of E7 oncoprotein, with the help of an HPV16 E7-targeting siRNA. E7 expression was significantly reduced in the cells transfected with HPV16 E7 siRNA, compared with the untransfected E7 expressing cells. TaqMan-based qRT-PCR analysis revealed 69% knockdown of HPV16 E7 expression in transfected group (T) compared to the untransfected (UT) (Fig. 3A). Corresponding Western blot analysis also revealed knockdown of E7 protein in transfected group (T) compared to the untransfected (UT) (Fig. 3B, Additional file 2: Figure S2).

Subsequently, we undertook chromatin immunoprecipitation (ChIP) followed by qRT-PCR assay, which revealed a significant depletion of H3K27me3 marks in E7 siRNA transfected SiHa cells (1.65-fold) in comparison to untransfected SiHa cells portraying E7 oncoprotein expression (573.8-fold) with a significance of *p* = 0.0009 (Fig. 3C-D). We employed EVX1 promoter primers as a positive control for H3K27me3 marks [31] as shown in Fig. 3C-D. Thus, HPV16 E7 oncoprotein appears to enhance the chromatin suppressive mark H3K27me3, at the MAL promoter region, which could be associated with EZH2 expression levels resulting in transcriptional suppression of the MAL gene.

Further, by employing qRT-PCR analysis, we found that E7 knocked-down SiHa cells showed significantly higher expression of MAL (1.6-fold, *p* = 0.03) (Fig. 3E) and lower expression of EZH2 (0.27-fold, *p* = 0.02) (Fig. 3F), a PRC2 complex member. EZH2 is the core catalytic subunit of PRC2 [32] bearing histone methyltransferase activity, which is known to introduce chromatin suppression mark H3K27me3. On the other hand, we did not observe any significant change in the expression level of the antisense lncRNA MAL-AS1, upon E7 knockdown in SiHa (Fig. 3G). Thus, even though E7 fails to affect the expression of lncRNA MAL-AS1, it acts to support cancer progression by lowering the level of MAL in CaCx through epigenetic modification at the MAL gene promoter. The finding evokes the need for interrogating the molecular interactions between E7, lncRNA AC103563.8 and EZH2.

### Interaction of AC103563.8 with viral oncoprotein E7 and cellular EZH2, at the same binding domain

We, thereby, undertook an *in silico* analysis employing catRAPID [33], a bioinformatic tool that predicts RNA–Protein interaction propensity and binding domains. This revealed a strong interaction propensity between oncoprotein E7 and AC103563.8 transcript and also between EZH2 protein and AC103563.8 transcript. E7 was found to bind to AC103563.8 at the nucleotide positions 294–479, with an interaction propensity of 50.23 and a discriminative power of 95%. On the other hand, EZH2 appeared to bind to AC103563.8 at nucleotide positions 284–478, with an interaction propensity of 85.62, and a discriminative power of 99%. Thus, it is evident that both E7 and EZH2 bear the potential of interacting with AC103563.8 at the same binding domain (Additional file 3: Figure S3A-B).

**Fig. 4:**
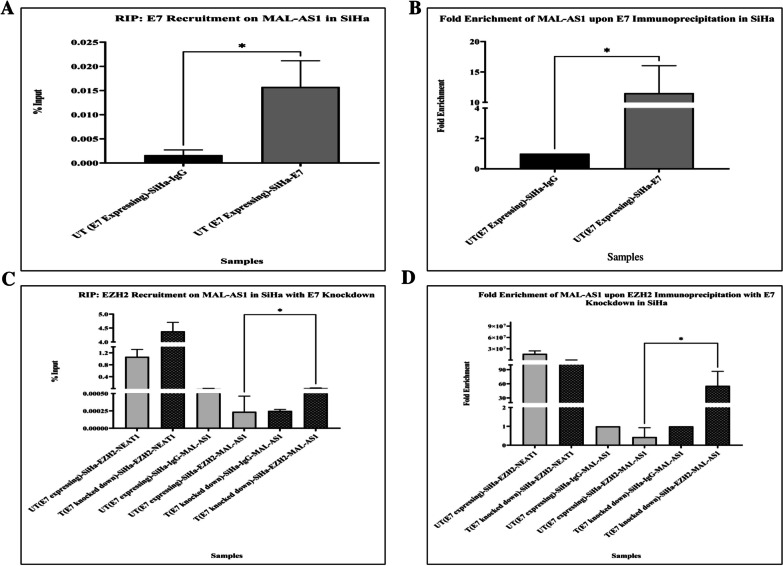
RNA immunoprecipitation (RIP)-qPCR with E7 and EZH2 antibody. **A** Percent input showing the enrichment of AC103563.8 in SiHa compared to that of IgG control upon E7 Immunoprecipitation. **B** Fold enrichment showing the enrichment of AC103563.8 in SiHa compared to that of IgG control upon E7 Immunoprecipitation. The data are represented as mean ± SD from two independent experiments. **C** Percent input showing the enrichment of AC103563.8 in transfected SiHa (T SiHa) compared to that of untransfected SiHa (UT SiHa) upon EZH2 Immunoprecipitation. **D** Fold enrichment showing the enrichment of AC103563.8 in transfected SiHa (T SiHa) compared to that of untransfected SiHa (UT SiHa) upon EZH2 Immunoprecipitation. NEAT1 is shown as the positive control for EZH2 interaction in UT and T SiHa. The data are represented as mean ± SD from two independent experiments

Subsequently, we confirmed the physical interaction of HPV16 E7 oncoprotein with AC103563.8 transcript, through RNA-Immunoprecipitation (RIP), with HPV16 E7 antibody in E7 expressing SiHa cells, followed by qRT-PCR assay. This showed an enrichment of AC103563.8 (11.51-fold, *p* = 0.0115), as compared to RIP with IgG antibody (negative control), in the HPV16-positive E7 expressing SiHa cells (Fig. 4A-B). On the other hand, a significant enrichment (*p* = 0.0361) of AC103563.8 was evident in EZH2 based immunoprecipitation, in E7-siRNA transfected SiHa cells, i.e. where HPV16 E7 was knocked down (55.5-fold) in comparison to EZH2 based immunoprecipitation in untransfected SiHa (0.43-fold), expressing E7 oncoprotein (Fig. 4C-D). We used NEAT1 promoter primers as a positive control for EZH2 interaction [34].

Such observations convincingly point towards the interplay between E7 oncoprotein, lncRNA AC103563.8 and EZH2, in regulating the MAL gene promoter epigenetically through chromatin suppressive H3K27me3 marks.

## Discussion

The underlying mechanisms that drive CaCx development involve extensive interactions between host molecules and HPV-encoded oncoproteins. Previous study from our laboratory [14] revealed the interaction of HPV16 E7 oncoprotein with lncRNA HOTAIR, which had an impact on PRC2-mediated global gene expression in such cancers, through epigenetic reprograming. Another study by McLaughlin-Drubin et.al. [35], on HPV16 E7 expressing cells and HPV16-positive cervical lesions, also revealed the impact of E7 resulting in enhanced expression of the homeobox genes KDM6A and KDM6B, through reduced H3K27me3 marks at the gene promoters. However, this study did not involve the role of any lncRNA in the process of gene regulation. A number of studies are however available on various cancer types that reveal the role of lncRNAs in gene regulation, through their involvement with chromatin regulating complexes or enzymes, resulting in histone modifications [36–39]. A very recent study [40], confirmed the presence of AC103563.8 as antisense of MAL in oral squamous cell carcinoma (OSCC). This study portrayed AC103563.8 as an oncogene that negatively regulated the expression level of MAL, which is downregulated in OSCC. AC103563.8 was found to inhibit the translation of MAL in a cis regulatory manner, thereby promoting invasion in OSCC. Our findings on HPV16-positive CaCx is contrary to the OSCC study findings [40] and those discussed above [36–39], as the presence of HPV16 E7 oncoprotein adds a new dimension, where both AC103563.8 and MAL are downregulated in CaCx.

Thus, taken together, our study and that of McLaughlin-Drubin [35] highlight the contextual epigenetic reprograming role of E7 in HPV16-positive cervical lesions, which prompted us to undertake the current study. Here, we aimed to decipher the interplay between host tumour suppressor molecule MAL, its novel antisense lncRNA AC103563.8 and HPV16 E7 oncoprotein and how their interaction associates with CaCx progression. Our study is the first report highlighting the mechanism of MAL gene expression downregulation in case of HPV16-related CaCx, or any other cancer types, involving enhanced chromatin suppression mark, H3K27me3 at the MAL promoter.

Based on the genomic location of lncRNA AC103563.8 in the vicinity of MAL and considering its genomic coordinates, this lncRNA appears to be antisense to MAL. Interestingly, it appears that AC103563.8, in association with HPV16 E7 oncoprotein, plays a major role in the manifestation of the chromatin suppressive H3K27me3 marks at the MAL promoter, suggestive of its regulatory potential of MAL gene expression. This is further supported by the significant positive correlated co-expression of both MAL and MAL-AS1 in such CaCx patients, both revealing downregulated expression and a higher correlative strength as opposed to that among the normal healthy individuals. It is well established that the regulatory potential of antisense RNAs, relate to their capacity of duplex formation with the RNAs transcribed from the complementary sense strands [41] to impact gene expression through various mechanisms such as DNA methylation [42], chromatin modification [43] and RNA degradation [44]. Our study clearly highlights that in the presence of E7, AC103563.8 acts to regulate the expression of MAL through chromatin modification. Therefore, perturbation of such correlated co-expression of this gene-pair by various means could be of potential therapeutic relevance.

There are reports available, which depict significant correlated co-expression of ncNATs with their sense coding genes [15] in cancers, compared to normal conditions. A significant proportion of such genes appear to be associated with patient prognosis in terms of survival, where the coding gene partner has often been identified to be associated with patient survival [15] and other adverse conditions. Besides, several genome-wide studies, a study employing nine cancer tissue types and strand-specific paired end RNA-seq data also confirmed positive correlation between a substantial number of antisense and sense transcripts in such cancers [17]. These observations render support to our observation in case of the antisense lncRNA AC103563.8 and MAL. However, little is known about the underlying mechanism associated with such observations in HPV-related CaCx. Therefore, we selected this gene pair (MAL and its antisense AC103563.8) out of several such gene pairs identified through our RNA seq analysis [19]. We were guided by the fact that MAL expression downregulation in various cancers has established MAL as a potent biomarker for cancer development in association with methylation of MAL promoter [22]. Clearly, we recorded that MAL gene expression downregulation was significantly associated with poor patient overall survival in our study, and also validated this observation in the TCGA CESC dataset. Our findings are in contrast to the findings from Alonso et. al. [22], who have reported lack of association between MAL expression and patient overall survival, considering KM plotter based *p* < 0.01, as the significant cut-off. In our study, we considered *p* < 0.05 as significant, as per standard practice. However, the MAL antisense lncRNA AC103563.8, alone failed to show such association with patient overall survival. Thereby it seems obvious that AC103563.8 acts to regulate MAL gene expression, thereby modulating patient survival, rather than portraying a direct effect.

MAL is known to function in membrane trafficking processes in polarised epithelial cells. Loss of epithelial cell polarisation is known to be one of the underlying mechanisms of neoplastic transformation of such cells, which results due to downregulated expression of MAL in such cells [29]. Thus, the MAL gene happens to be a robust candidate tumour suppressor gene [45]. Downregulated expression of MAL has been demonstrated in several cancers of epithelial origin [29, 46], including cervical cancers [45, 47], justifying its role as a tumour suppressor.

There are evidences which suggest that DNA hypermethylation and H3K27me3 go hand in hand in cancer [48]. A study on various astrocytic tumours [49], employed genome wide ChIP-seq analysis for H3K27me3 modification to reveal that a large majority of H3K27me3 target genes were downregulated in association with DNA methylation, apart from H3K27me3 marks. Whether a similar phenomenon prevails in case of HPV16-positive CaCx or cell-lines, is yet to be reported. However, preliminary data (unpublished) from our laboratory based on 850 K Infinium Methylation EPIC Bead Chip assay on HPV16-positive CaCx patients (*n* = 49) and HPV negative normal healthy individuals (*n* = 27), revealed hypermethylation in the promoter region of MAL among CaCx patients. This sample set, both patients and normal individuals, were inclusive of the sample set analysed for MAL gene expression in our current study. However, this finding remains to be validated. Nevertheless, the possibility of coexistence of H3K27me3 marks and DNA methylation in the MAL gene promoter manifesting in downregulated expression of MAL in our patient samples, cannot be ruled out.

**Fig. 5:**
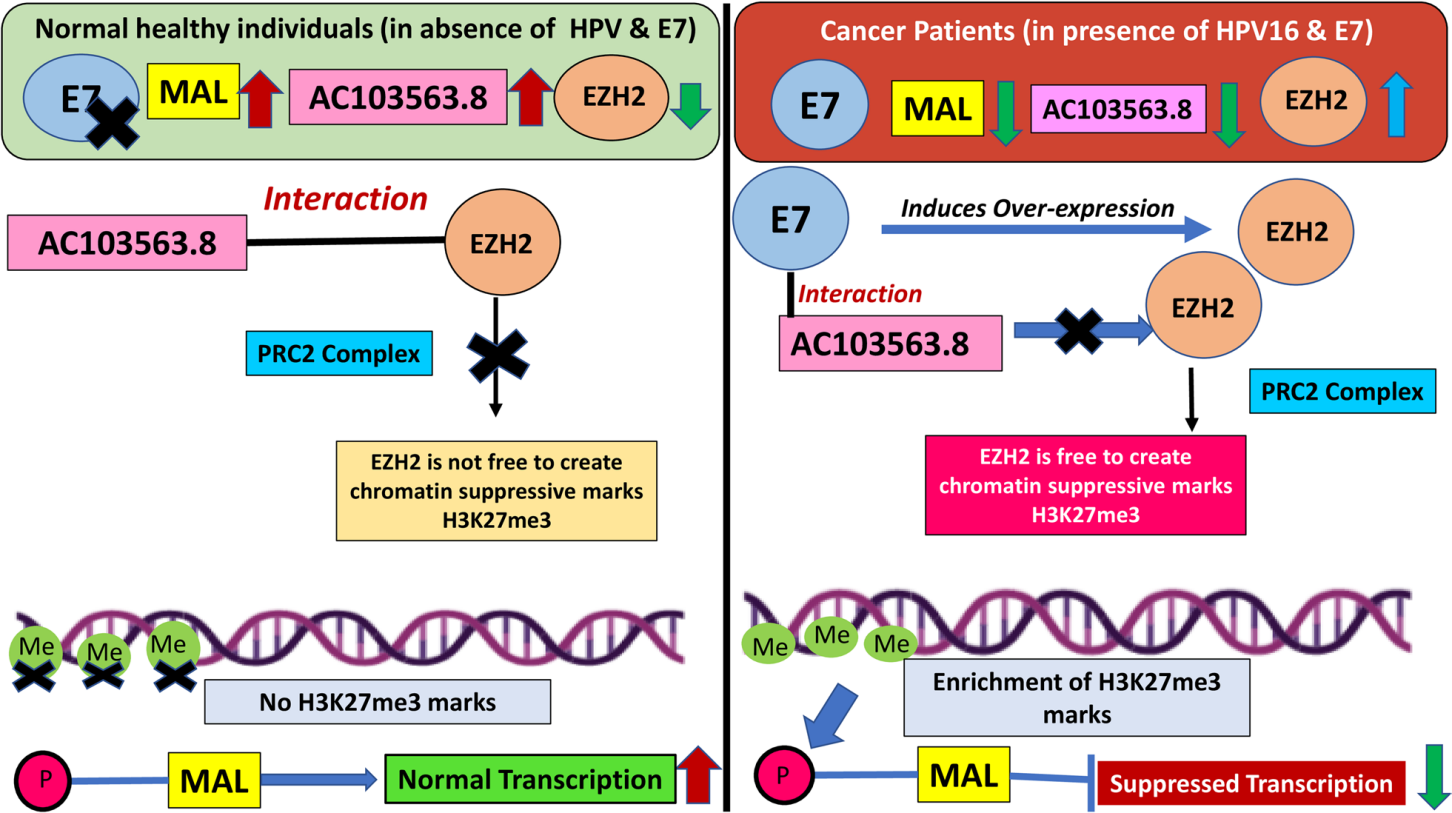
Hypothetical functional model. Interplay of HPV16-E7/AC103563.8/EZH2/MAL axis in CaCx pathogenesis

Since host–pathogen interaction is a prerequisite in CaCx and based on association of E7 induced enhanced H3K27me3 with MAL expression downregulation in the HPV16-positive SiHa cells, we stepped forward to unfurl the complex mechanism driving this phenomenon. We observed that HPV16 E7 failed to influence the expression of AC103563.8, but maintained high levels of EZH2 expression. The latter observation was based on the fact that EZH2 level was significantly downregulated under the condition of E7 knockdown in SiHa cells. This is further supported by earlier observation from our laboratory [14], where we recorded an upregulated expression of EZH2 under the condition of E7 overexpression in C33A cells. But E7 was found to interact with AC103563.8, at the same binding domain as that of EZH2. Thus, in the presence of E7, among the CaCx cases, AC103563.8, by virtue of binding with E7, fails to interact with EZH2. Therefore, EZH2 appears to be free to create the chromatin suppression mark H3K27me3 at the MAL promoter, resulting in downregulated expression of MAL supporting poor patient survival. Contrary to this, in a hypothetical situation, i.e. in the absence of HPV16 infection and E7 expression among normal healthy individuals, AC103563.8 is likely to interact with EZH2 and preclude it from the PRC2 complex. This could thereby prevent the creation of H3K27me3 marks at the MAL promoter, supporting the normal transcription of MAL. This is summarised in our proposed hypothetical functional model (Fig. 5).

## Conclusion

We have successfully confirmed through functional analysis combined with strand specific RNA-seq that the MAL antisense lncRNA AC103563.8 is an important regulator of its sense gene MAL, which is significantly associated with CaCx patient prognosis. Therefore, we propose that it may be termed as MAL-AS1. Most importantly, our study highlights the uniqueness of HPV16-related CaCx, where HPV16 E7 oncoprotein plays a key role in decoupling the interaction between AC103563.8 (MAL-AS1) and the PRC2 complex member EZH2, facilitating the enhancement of H3K27me3 marks at the MAL promoter to suppress MAL expression. Interestingly, the host antisense lncRNA molecule, AC103563.8 (MAL-AS1), appears to interact with both the host-encoded EZH2 and the viral oncoprotein E7. Thus, the MAL-AS1/E7/EZH2 axis seems to be crucial in the expression regulation of the survival-associated gene, MAL, through chromatin inactivation in HPV16-related CaCx pathogenesis, warranting therapeutic strategy development.

## Materials and methods

### Subjects, samples and cell lines

We collected normal cervical tissue biopsies from married women, aged 28–50 years (median age: 43 years), who underwent hysterectomy for reasons other than cancer and the cancer biopsies from women aged 35–78 years (median age: 54 years) who were diagnosed with CaCx at the collaborating hospitals. Every individual, who provided us with a tissue sample also provided a written informed consent that was in conformity with the approved institutional ethical guidelines. We also collected the histopathology reports of all samples. We have provided the details on sample processing, sequencing and identification of differentially expressed genes in the Additional file 4. We considered HPV16-positive CaCx cell line SiHa for further experiments.

### Visualisation and correlation between MAL and AC103563.8

For the visualisation of the highly downregulated gene pair, MAL and AC103563.8, we used the pheatmap R package (https://cran.r-project.org/web/packages/pheatmap/pheatmap.pdf) to perform unsupervised hierarchical clustering with normalised counts, which were log2 transformed after adding a constant (= 1) to all values. We then calculated the Pairwise Pearson’s correlation coefficient (*r*) between MAL and AC103563.8 using their normalised expression, i.e. TPM values. Then, we generated the Correlation plot using R package (corrplot). Subsequently, we performed differential correlation analysis of the gene pair between cancer patients and healthy individuals using Fisher’s z transformation of r (http://vassarstats.net/rdiff.html).

### Overall patient survival analysis

We determined the association of patient survival with MAL expression deregulation, with TCGA Cervical squamous cell carcinoma and endocervical adenocarcinoma (TCGA-CESC) dataset, using KM plotter (https://kmplot.com/analysis/) [50]. This dataset included 304 CaCx samples.

### Determination of gene expression (mRNA) by real-time PCR in cervical tissues and cell line SiHa

We employed about 400 ng of the isolated total RNA from normal (*n* = 19) and HPV16-positive CaCx (*n* = 22) tissues and SiHa cell line (untransfected SiHa and E7 knocked down SiHa) to prepare cDNA using combinations of oligo-dTP3 and random hexamers with “High-capacity cDNA reverse Transcription Kit” of Applied Biosystems (Cat. No. 4368814, Thermo Scientific, USA) on ABI-Quant Studio 5. Then we used the cDNA to estimate the expressions of MAL, AC103563.8, and EZH2, using SYBR-green based qRT-PCR with Glyceraldehyde 3-phosphate dehydrogenase (GAPDH) as internal control. The primer sequences, amplicon sizes and PCR conditions employed are available in Additional file 5: Table S1. We calculated the fold change by 2^(−∆∆Ct)^ method. The details of all the amplification and dissociation curves from real-time PCR are available in Additional file 6: Figure S4.

### Cell culture, knockdown of HPV16 E7 in SiHa cell line, quantification of E7 mRNA by TaqMan-based real-time PCR and detection of E7 oncoprotein by Western blot analysis

We cultured the SiHa cells adhering to the ATCC protocols, as described earlier from our laboratory [51]. We knocked down HPV16E7 in SiHa using siRNA against HPV16 E7 (Cat. No. sc-270423, Santacruz, USA) with non-targeted siRNA (Eurogentec, Cat. No. SR-CL000-005, Belgium) as negative control. We seeded about 3 × 10^6^ SiHa cells in 10 cm culture plates and after 24 h, we transfected the cells with 80 pmol of HPV16 E7 siRNA (transfected) and control siRNA (negative control) using Lipofectamine 2000 (Invitrogen, Cat. No. 11668019, USA). We harvested and washed the cells after 48 h of transfection, with 1 × PBS (pH 7.4), trypsinised, and collected them by centrifugation at 300 g for 10 min. We used the transfected cells further for RNA isolation. We carried out the transfection experiments in three sets. We also isolated the RNA and protein for HPV16 E7 expression analysis.

We isolated the RNA by Trizol method [52] and prepared the cDNA following similar protocols as mentioned above. We performed real-time PCR using the cDNA to estimate the expression of HPV16 E7 by TaqMan-based assay (Cat.No. N8080234, Thermo Scientific, USA). We considered 18srRNA as internal house-keeping gene and the assay was performed employing QuantStudio 5 (Thermo Scientific, USA). We calculated the fold change by 2^(−∆∆Ct)^ method.

For protein assay, we lysed the cells using RIPA lysis buffer and quantified the protein concentration using Bradford assay. We separated about 30–50 μg of proteins by 15% SDS-PAGE and then transferred to PVDF membrane, followed by membrane blocking with 5% skimmed milk, incubation with primary antibody for HPV16 E7 (Cat. No. sc-6981, Santacruz, USA) at 1:500 dilution overnight, at 4 °C and incubation with HRP-conjugated secondary antibody (Cat. No. ab205719, Abcam, UK) at 1:5000 dilution. We considered β-actin (Cat. No. ab6276, Abcam, USA) as loading control. Finally, we visualised the protein bands using ECL substrate Kit (Cat. No. 1705062, BioRad ECL max kit, USA).

### Chromatin immunoprecipitation (ChIP)-qPCR

We crosslinked the unperturbed SiHa cells and SiHa cells with E7 knockdown, with 37% formaldehyde and incubated at room temperature (RT) for 10 min. We quenched the formaldehyde by adding glycine and incubated at RT for 5 min. We collected the cells in ice-cold PBS along with freshly added 1X Protease inhibitor (PI) and we snap froze the cells in Liquid Nitrogen. We further lysed the cells using RIPA lysis buffer and fragmented the chromatin to 200–800 bp sizes using Covaris S220 (USA). The ChIP’d DNA showed a fragment size ranging between 50 bp–1.5 Kb as identified by agarose gel electrophoresis (Additional file 7: Figure S5). After centrifugation, we diluted about 50 μl of the supernatant to 500 μl with dilution buffer containing freshly added PI. We added H3K27me3 antibody (Cat. No. ab6002, Abcam, USA) and IgG (Cat.No. ab205719, Abcam, USA) at 2 μg for each, and 30 μl of A/G magnetic beads to the chromatin solution and the mixture was incubated at 4 °C overnight. We sequentially washed the immunoprecipitated samples using low salt, high salt, LiCl and TE wash buffers and finally eluted with elution buffer, along with Proteinase K incubation at 65 °C for 4 h. We collected the supernatant and purified the DNA using QIAquick Gel Extraction kit (Cat. No. 28704, Qiagen, Germany). We also purified the DNA from input control sample, which did not undergo any immunoprecipitation. We analysed the enrichment of H3K27me3 marks by qRT-PCR using input DNA and IgG pull-down ChIP DNA as controls at the MAL promoter region. We used EVX1 as the positive control for H3K27me3 marks. The primer sequences are available in Additional file 5: Table S1.

### In silico tool catRAPID based determination of RNA–protein interaction

We performed the *in silico* prediction to identify possible interaction between antisense AC103563.8 transcript and HPV 16 E7 oncoprotein and between AC103563.8 transcript and EZH2 protein, using the “catRAPID fragments” in the catRAPID predictor tool (http://service.tartaglialab.com/page/catrapid_group) [33]. We confirmed the identified interactions through RNA Immunoprecipitation and qPCR, as described below.

### RNA immunoprecipitation (RIP)-qPCR

We collected the transfected SiHa (T) cells (E7 knocked-down with siRNA as described earlier) and the untransfected SiHa (UT) cells (unperturbed and expressing E7), in ice-cold PBS with freshly added 200X PI and then snap froze these cells in Liquid Nitrogen. We then lysed the cells using RIPA lysis buffer, PI, and RNAase inhibitor (RI) cocktail. After centrifugation, we proceeded with 100 μl of supernatant for the immunoprecipitation assays. We performed the DNAase treatment of these supernatants. We added the RIP dilution buffer (9 times the volume of each supernatant) along with PI and RI. Then we added the antibodies (E7 on UT, EZH2 on UT and T, IgG on UT and T) at 5 μg of each, along with 50 μl of A/G magnetic beads to each mixture and incubated those at 4 °C, overnight. Thereafter, we sequentially washed the immunoprecipitated (IP) samples using EZH2 antibody (Cat. No. 5246, Cell Signalling Technology, USA) and IgG antibody as the negative control, using low salt, high salt, LiCl and TE wash buffers and finally eluted with elution buffer along with Proteinase K and 10% SDS at 60 °C for 30 min. We collected the supernatant and isolated the RNA using the Trizol method [52]. We purified the RNA from input control samples, which did not undergo any immunoprecipitation. We analysed the expression of AC103563.8 transcripts within E7 and EZH2 IP samples by qRT-PCR using input RNA and IgG pull-down RIP RNA, as controls. We used NEAT1 as a positive control for EZH2 interaction. We designed the AC103563.8 primer sequences from its binding domains with E7 and EZH2, which is provided in Additional file 5: Table S1.

### Statistical analysis

We used the GraphPad Prism (version 9.5.1, San Diego, CA) software for statistical analyses of data. We analysed the gene expression data (based on real-time qRT-PCR of tissue samples) using the Wilcoxon signed ranked test (nonparametric), after testing for normality based on Kolmogorov–Smirnov test. For multiple sets of cell line-based experiments, we compared using the two-tailed Student’s t-test for statistically significant differences in expression levels between the experimental categories. As indicated, we considered *p* < 0.05 as significant and represented the significance levels as: *p < 0.05; **p < 0.01; ***p < 0.001; ****p < 0.0001; ns, not significant (*p* > 0.05).

### Supplementary Information

**Additional file 1: Fig. S1. **Depiction of the location DEG pair: MAL- AC103563.8 on chromosome 2. Ensembl database showing the genomic location of MAL and its antisense lncRNA AC103563.8 and the coordinates of the corresponding transcripts.

**Additional file 2: Fig. S2.** Western blot. Full Western blot analysis of (**A**) HPV16E7 (19 KDa) in UT-SiHa (untransfected SiHa) and transfected SiHa (T-SiHa). (**B**) Same blot stripped and Western blot performed for house-keeping protein β-actin (42 KDa) for UT-SiHa (untransfected SiHa) and T-SiHa (transfected SiHa).

**Additional file 3: Fig. S3.** In silico analysis reveals AC103563.8 interaction with oncoprotein E7 and EZH2 protein. “catRAPID fragments” based prediction of interaction between (**A**) AC103563.8 and HPV16 E7 (**B**) AC103563.8 and EZH2. X-axis indicates the nucleotide position, i.e. the nucleotide position of AC103563.8 where the proteins E7 and EZH2 bind to it. Y-axis represents the interaction Z score.

**Additional file 4. Supplementary Methods.** (i) Sample processing and sequencing, and (ii) Alignment of RNA-sequence data and identification of differentially expressed genes, discussed in details [53–57].

**Additional file 5: Table S1.** Table representing the sequences of the primers used, PCR program and product lengths.

**Additional file 6: Fig. S4.** Amplification and Dissociation curves of real-time PCR reactions.

**Additional file 7: Fig. S5.** Chromatin immunoprecipitation DNA fragmentation. Sheared chromatin fragments for chromatin immunoprecipitation for UT-SiHa (untransfected SiHa) and T-SiHa (transfected) with size ranging from 50 bp to 1.5 Kb.

## Data Availability

Raw RNA seq BAM files of individual patients can be accessed from European Nucleotide Archive (ENA) via accession number: PRJEB40877 and secondary accession number: ERP124576. Raw data will be made available upon request.
