# Supplementary figures and images for: MAL expression downregulation through suppressive H3K27me3 marks at the promoter in HPV16-related cervical cancers is prognostically relevant and manifested by the interplay of novel MAL antisense long noncoding RNA AC103563.8, E7 oncoprotein and EZH2
Source: Clin Epigenetics. 2024 Mar 10;16:40. doi: 10.1186/s13148-024-01651-9 (PMC10924967; doi:10.1186/s13148-024-01651-9)

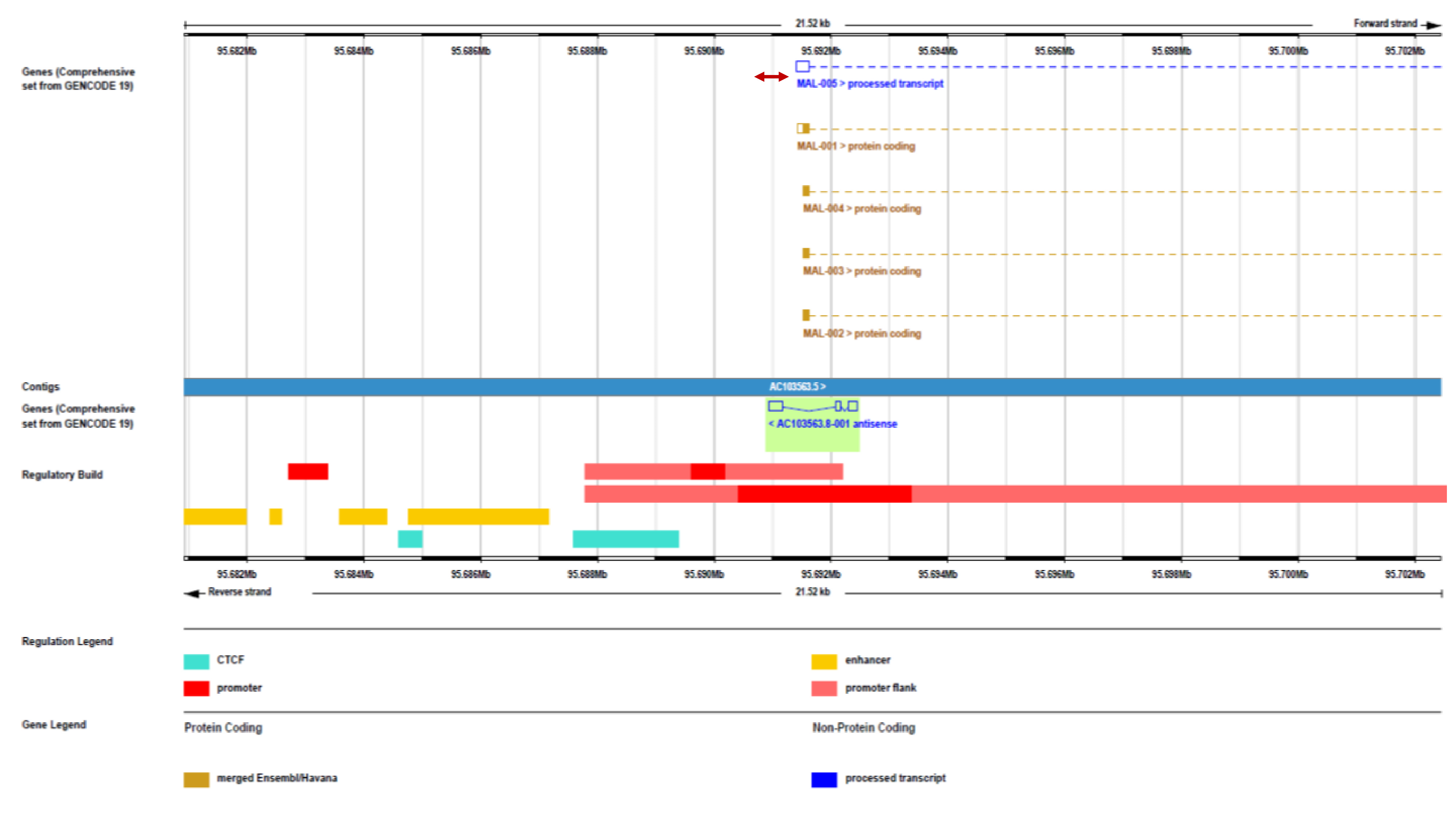

Supplement: Supplementary file 1 — Additional file 1: Fig. S1. Depiction of the location DEG pair: MAL- AC103563.8 on chromosome 2. Ensembl database showing the genomic location of MAL and its antisense lncRNA AC103563.8 and the coordinates of the corresponding transcripts. [file 13148_2024_1651_MOESM1_ESM.tif]

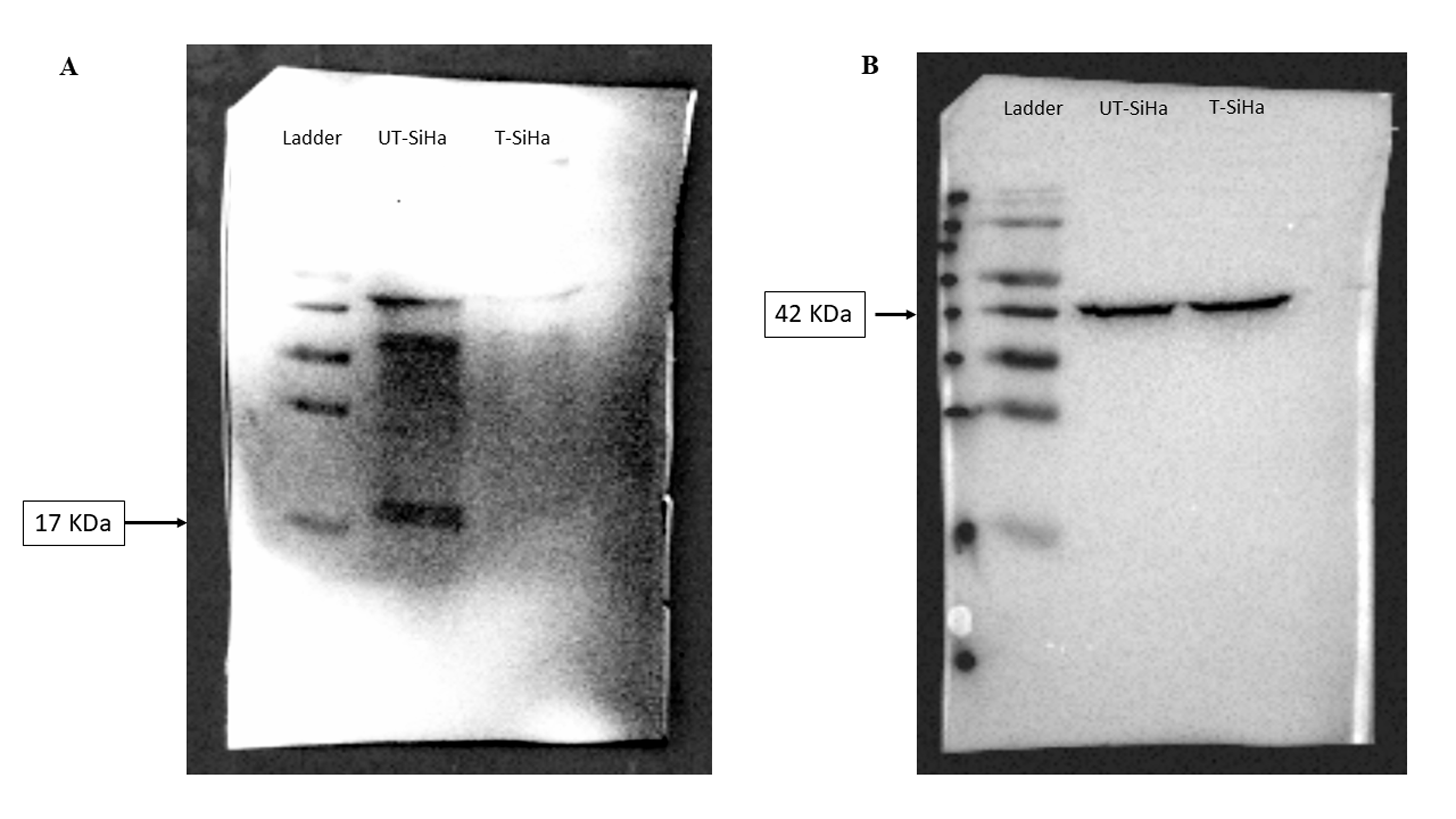

Supplement: Supplementary file 2 — Additional file 2: Fig. S2. Western blot. Full Western blot analysis of (A) HPV16E7 (19 KDa) in UT-SiHa (untransfected SiHa) and transfected SiHa (T-SiHa). (B) Same blot stripped and Western blot performed for house-keeping protein β-actin (42 KDa) for UT-SiHa (untransfected SiHa) and T-SiHa (transfected SiHa). [file 13148_2024_1651_MOESM2_ESM.tif]

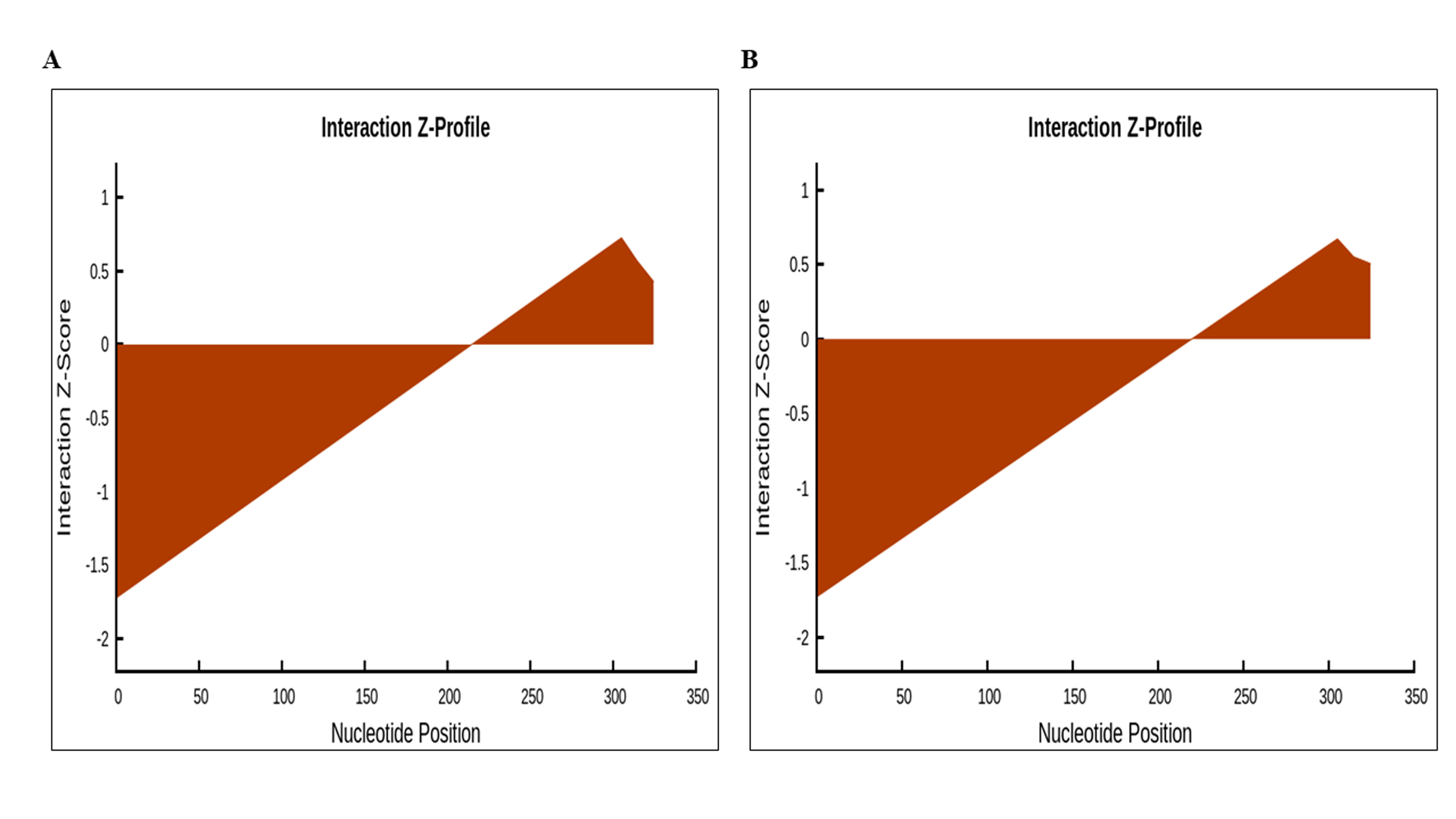

Supplement: Supplementary file 3 — Additional file 3: Fig. S3. In silico analysis reveals AC103563.8 interaction with oncoprotein E7 and EZH2 protein. “catRAPID fragments” based prediction of interaction between (A) AC103563.8 and HPV16 E7 (B) AC103563.8 and EZH2. X-axis indicates the nucleotide position, i.e. the nucleotide position of AC103563.8 where the proteins E7 and EZH2 bind to it. Y-axis represents the interaction Z score. [file 13148_2024_1651_MOESM3_ESM.tif]

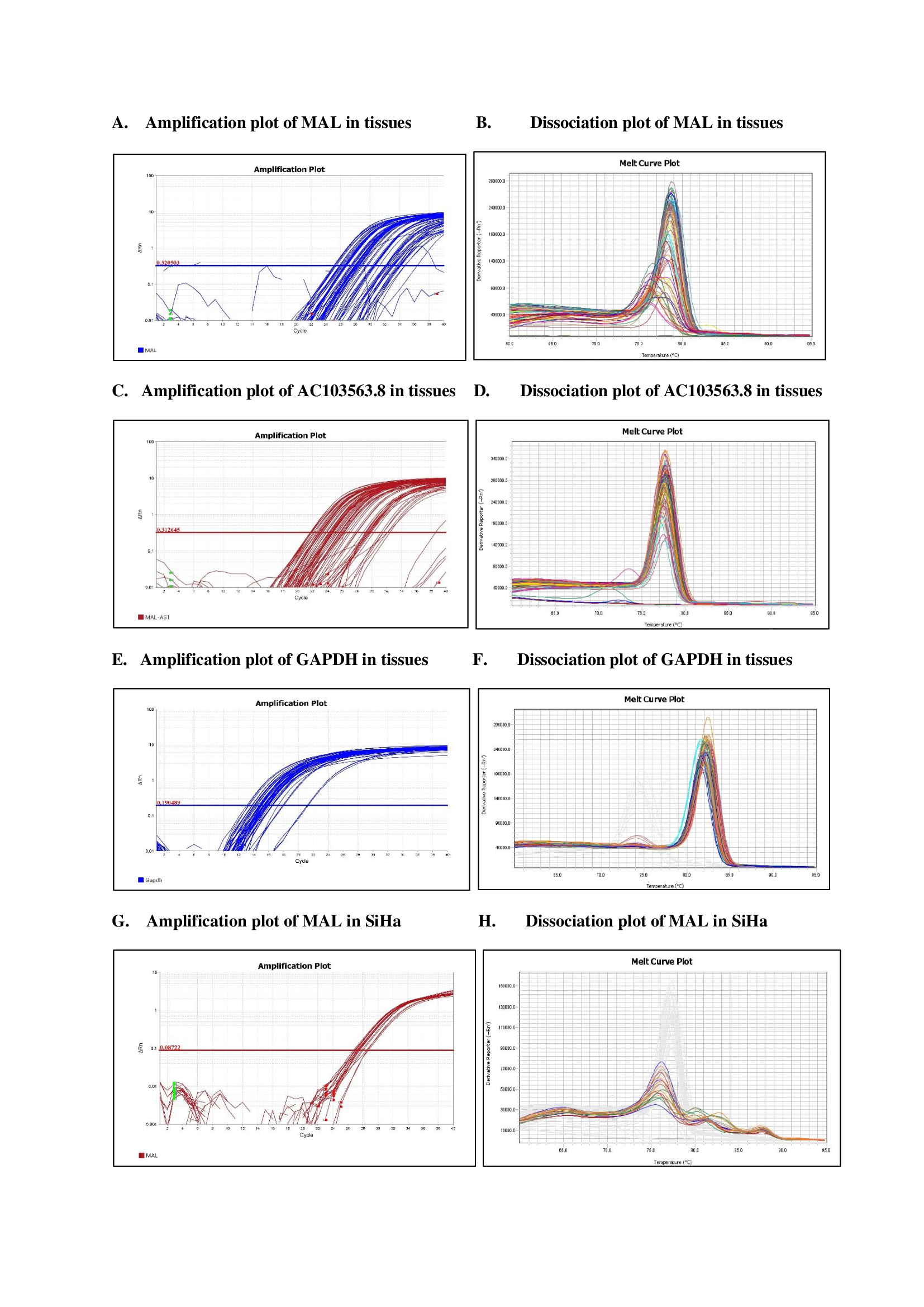

Supplement: Supplementary file 6 — Additional file 6: Fig. S4. Amplification and Dissociation curves of real-time PCR reactions. [file 13148_2024_1651_MOESM6_ESM.tiff]

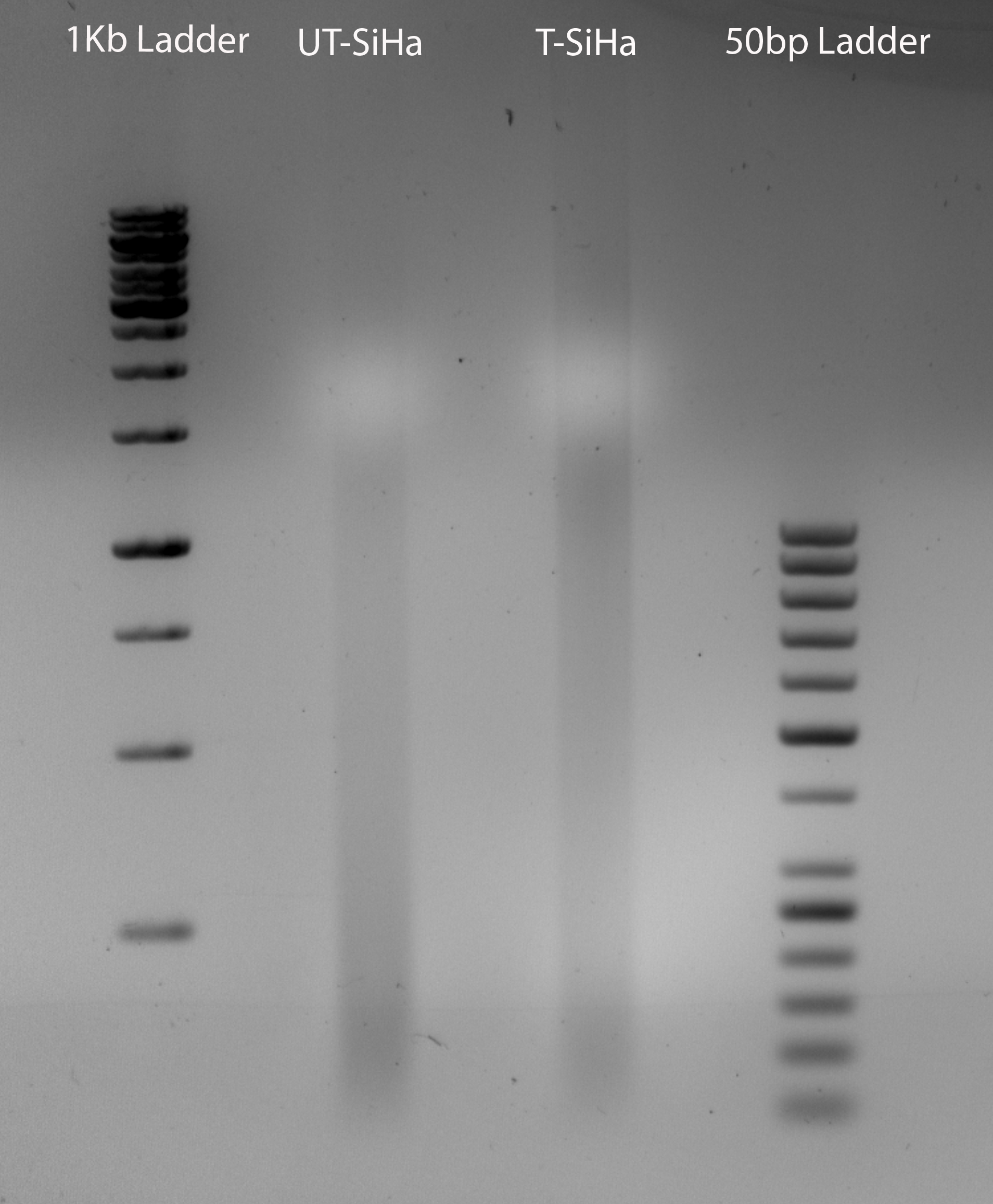

Supplement: Supplementary file 7 — Additional file 7: Fig. S5. Chromatin immunoprecipitation DNA fragmentation. Sheared chromatin fragments for chromatin immunoprecipitation for UT-SiHa (untransfected SiHa) and T-SiHa (transfected) with size ranging from 50 bp to 1.5 Kb. [file 13148_2024_1651_MOESM7_ESM.tiff]
