# Supplementary material for: MAL expression downregulation through suppressive H3K27me3 marks at the promoter in HPV16-related cervical cancers is prognostically relevant and manifested by the interplay of novel MAL antisense long noncoding RNA AC103563.8, E7 oncoprotein and EZH2
Source: Clin Epigenetics. 2024 Mar 10;16:40. doi: 10.1186/s13148-024-01651-9 (PMC10924967; doi:10.1186/s13148-024-01651-9)
Supplement: Supplementary file 4 — Additional file 4. Supplementary Methods. (i) Sample processing and sequencing, and (ii) Alignment of RNA-sequence data and identification of differentially expressed genes, discussed in details [53–57]. [file 13148_2024_1651_MOESM4_ESM.docx]

**Supplementary Methods**

***Sample processing and sequencing***

We collected the cervical tissue biopsies in RNAlater. We isolated the genomic DNA and total RNA from these tissues using QIAamp DNA mini kit and RNeasy mini kit (Qiagen) respectively, following the manufacturers protocol with some modifications. We performed the quality check of the RNA using Agilent Bioanalyzer 2100. Details regarding DNA isolation, HPV screening and HPV type identification, are described in details in our earlier studies (14,53). We tested all the samples for the presence of HPV and classified as HPV-negative or positive. Of the HPV positive samples, we selected only those with HPV16 infection as HPV16 is the most prevalent type in CaCx cases in India. We excluded the samples showing presence of both HPV18 and HPV16 from the study. For this study, we compared the HPV-negative normal samples (n=34) with HPV16-positive CaCx samples (n=44). We used the TruSeq Stranded Total RNA Library Prep kit (Illumina) for library preparation. We assessed the library quality using Agilent Bioanalyzer 2100 system and then sequenced employing Nova-Seq 6000 (Illumina) to generate paired-end reads of 100 bases.

***Alignment of RNA sequencing data and identification of differentially expressed genes***

We adopted a community standard for analysis that included using FastQC-0.11.7 (http://www.bioinformatics.bbsrc.ac.uk/projects/fastqc/), STAR aligner (STAR-2.6.0c) (54) HTSeq (HTSeq-0.11.0) (55) and DESeq2 1.22.2 with R version 3.5.1(56). For our analysis, we considered only the genes for which, there was significant (p<0.05 after correction using the Benjamini-Hochberg [BH] procedure) differential expression and a high expression fold-change (FC), |log2(FC)| ≥ 2. TPM (transcripts per million). We calculated the TPM values using the TPMCalculator tool_0.0.3 (57). For identification of long noncoding genes, especially antisense transcripts and their coding partners, we restricted to considering only those transcripts that were of length > 200 nucleotides, as identified from LNCipedia version 5.2 <https://lncipedia.org/db/search> and Ensembl (<https://asia.ensembl.org/index.html>) database.
