# Supplementary material for: MAL expression downregulation through suppressive H3K27me3 marks at the promoter in HPV16-related cervical cancers is prognostically relevant and manifested by the interplay of novel MAL antisense long noncoding RNA AC103563.8, E7 oncoprotein and EZH2
Source: Clin Epigenetics. 2024 Mar 10;16:40. doi: 10.1186/s13148-024-01651-9 (PMC10924967; doi:10.1186/s13148-024-01651-9)
Supplement: Supplementary file 5 — Additional file 5: Table S1. Table representing the sequences of the primers used, PCR program and product lengths. [file 13148_2024_1651_MOESM5_ESM.docx]

**Supplementary Table S1:** Table representing the sequences of the primers used, PCR program and product lengths.

**Primer details of qPCR**

| **Gene ID** | **Primer Sequence** | **Product Length** |
| --- | --- | --- |
| MAL qPCR | F: GCAAGACGGCTTCACCTACAC  R: GCAGAGTGGCTATGTAGGAGAACA | 74 |
| AC103563.8 qPCR | F: CAAGATCGAATTGGTCGCAAATA  R: CACCGAGATTTGGAGATGAGAG | 101 |
| GAPDH qPCR | F: GTCTCCTCTGACTTCAACAGCG  R: ACCACCCTGTTGCTGTAGCCAA | 131 |
| EZH2 | F: CCC TGA CCT CTG TCT TAC TTG TGG A  R: CGT CAG ATG GTG CCA GCA ATA | 119 |
| AC103563.8-RIP | F: GACACCGTGAGCGCTTATCA  R: GTTTCTGGCGAGGGAACGTA | 281 |
| MAL-Promoter-ChIP | F: CACATTAACGCATCCAGCGG  R: CTCATCAAGGCACCTCTCGG | 83 |
| EVX1-Promoter | F: GCTACTGCCTTCTCCCAAGT  R: GAAGTCGCTTTCCCGTTTGC | 222 |
| NEAT1 | F: CTTCCTCCCTTTAACTTATCCATTCAC  R: CTCTTCCTCCACCATTACCAACAATAC | 116 |
| E7-qPCR | F: AAGTGTGACTCTACGCTTCGGTT  R: GCCCATTAACAGGTCTTCTTCCAAA | 78 |

**PCR Program Details**

| **PCR program for E7 estimation** |
| --- |
| UNG-activation- 50°C (2 minutes)  Initial denaturation-95°C (10 minutes)  Denaturation-95°C (15 seconds) 40 Cycles  Annealing-60°C (1 minute) |

| **PCR program for all other gene expressions except ChIP** |
| --- |
| UNG-activation- 50°C (2 minutes)  Initial denaturation-95°C (10 minutes)  Denaturation-95°C (15 seconds) 40 Cycles  Annealing-60°C (1 minute)  Initial denaturation-95°C (15 seconds)  Annealing-60°C (1 minute) Melt Curve  Dissociation-95°C (15 seconds) |

| **PCR program for ChIP** |
| --- |
| UNG-activation- 50°C (2 minutes)  Initial denaturation-95°C (10 minutes)  Denaturation-95°C (15 seconds) 50 Cycles  Annealing-60°C (1 minute)  Initial denaturation-95°C (15 seconds)  Annealing-60°C (1 minute) Melt Curve  Dissociation-95°C (15 seconds) |
